# Chemical Interference: A Review on Endocrine Disruptors and Reproductive Communication in Amphibians

**DOI:** 10.1002/ece3.71986

**Published:** 2025-08-25

**Authors:** Martyna Frątczak, Mikołaj Kaczmarski, Katarzyna Szkudelska, Piotr Tryjanowski

**Affiliations:** ^1^ Department of Zoology Poznan University of Life Sciences Poznan Poland; ^2^ Department of Animal Physiology, Biochemistry and Biostructure Poznan University of Life Sciences Poznan Poland

**Keywords:** acoustic communication, agrochemicals, hormones, olfactory communication, pheromones, pollution

## Abstract

Amphibians are highly vulnerable to anthropogenic pollution, primarily due to their permeable skin and eggs, as well as their habitat preferences. Endocrine‐disrupting compounds (EDCs), prevalent in aquatic environments and soil, pose a significant threat to their survival. While the physiological effects of EDCs on amphibians have been extensively studied, their impact on behavior remains relatively unexplored. This paper reviews the existing literature on the impact of EDCs on the mating behavior of amphibians, including disruptions in acoustic, olfactory, and visual communication. Although it has been shown that amphibian reproduction can be affected by endocrine disruptors, there are still significant research gaps. We performed an extensive review of the literature, which yielded only 27 eligible studies—21 of which tested the effects on mating communication and behavior, and only 6 examined the impact on body coloration. There is a strong need for a deeper understanding of how EDCs, both alone and in combination with other stressors, affect the reproductive behavior of amphibians, as this may have serious implications for the dynamics and survival of entire populations and species.

## Graphical abstract

Amphibians are particularly susceptible to anthropogenic pollution. This paper reviews how endocrine‐disrupting compounds (EDCs) affect their mating behavior, noting significant research gaps in understanding these impacts. Further studies are crucial to grasp EDCs' effects on amphibian reproduction and population dynamics.

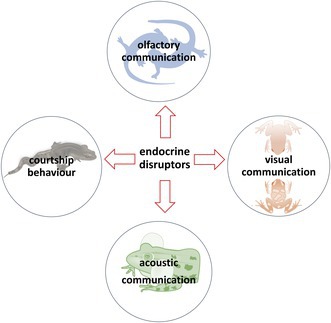

## 1 Introduction

Amphibians are a group of vertebrates particularly sensitive to anthropogenic changes, which threaten their survival globally (Luedtke et al. 2023). They are highly susceptible to contaminants due to their permeable eggs and skin, as well as their habitat preferences. Most species develop in aquatic environments (Wells 2008), whereas adult individuals often reside in areas prone to anthropogenic pollution such as agricultural lands (Băncilă et al. 2023; Brühl et al. 2011, 2013), wetlands (Sievers, Hale, Parris, et al. 2019) and forests (Chen et al. 2004; Lambert and Skelly 2016). Numerous contaminants found in these habitats, including pesticides, fertilizers, heavy metals, and plasticizers, are recognized as endocrine‐disrupting compounds (EDCs) and are known to impact amphibian growth, development, and survival (Baker et al. 2013; Bókony et al. 2018; Goto et al. 2006; Lefcort et al. 1998; Papoulias et al. 2013; Wojtaszek et al. 2004). Additionally, the food base of amphibians in these areas, which often includes invertebrates and small vertebrates, can be a source of EDCs (González‐Alcaraz et al. 2020; Markman et al. 2007).

The impact of EDCs on amphibian physiology has already been extensively studied (Gyllenhammar 2008; Hayes et al. 2002; Jagnytsch et al. 2006; Kloas 2002; Tamschick, Rozenblut‐Kościsty, Ogielska, Kekenj, et al. 2016; Tamschick, Rozenblut‐Kościsty, Ogielska, Lehmann, et al. 2016). A significant number of studies focus on the impact of EDCs on the reproductive system of amphibians, in particular the anatomy and histology of gonads (for the review, see Frątczak et al. 2025). Among the most striking are cases of mixed‐sex conditions or complete sex reversal (e.g., Cai et al. 2020; Chardard et al. 2003; Oka et al. 2006; Tamschick, Rozenblut‐Kościsty, Ogielska, Kekenj, et al. 2016; Tamschick, Rozenblut‐Kościsty, Ogielska, Lehmann, et al. 2016). However, the impact of EDCs on amphibian behavior—especially that related to communication, mating, and reproduction—remains far less explored (Sievers, Hale, Parris, et al. 2019; Orton et al. 2023).

In many amphibian species, reproductive behaviors rely heavily on communication between mates, involving a series of hormonally regulated actions such as mate location, courtship, and mating. These behaviors are often sexually dimorphic and mediated through acoustic, olfactory, and visual signals, depending on the species and ecological context (Sever and Staub 2011; Woodley 2011). For example, males of most anuran species produce advertisement calls to attract females and to signal sexual readiness (Wells and Schwartz 2006), while in many Caudata, pheromonal cues play a key role in mate attraction and stimulation (Kikuyama et al. 2002). In some taxa, visual traits such as body coloration (Bell 2005; Rojas et al. 2023) or limb movements, e.g., arm or tail waving (Halliday 1990), may serve as supplementary courtship signals. These traits are under endocrine control and may be potentially disrupted by exposure to EDCs (Orton et al. 2023), affecting mating success and, ultimately, reproductive output. These effects can have profound consequences on the survival of the populations and whole species of amphibians.

Therefore, understanding how EDCs affect amphibian behavior is essential for recognizing their true ecological impact. Taking this into account, we review the effects of EDCs on amphibians, focusing on their impact on reproductive behavior through disruptions of acoustic, olfactory, and potentially visual communication. We aim to highlight the existing research gaps and encourage greater interest in this topic.

## 2 Endocrine Disruptors and Their Behavioral Implications

EDCs are substances that mimic or interfere with the endogenous hormones of organisms (European Commission 1996). The majority of EDCs found in the environment are of anthropogenic origin and represent a diverse group of compounds, including pesticides, plasticizers, surfactants, pharmaceuticals, and residues from personal care products. EDCs have the capacity to bind to hormone receptors, particularly affecting estrogenic, androgenic, and thyroid signaling pathways (La Merrill et al. 2020). They have been shown to influence sexual characteristics across all vertebrate groups (Kloas and Lutz 2006) and are associated with numerous reproductive disorders in animals (Ghosh et al. 2022).

In recent years, there has been growing awareness that EDCs can also modify behavior across a wide range of species (Clotfelter et al. 2004). Exposure to EDCs has been linked to altered reproductive behaviors in mammals, including humans (Gore et al. 2019; López‐Rodríguez et al. 2021; Patisaul and Adewale 2009; Rosenfeld 2015). Additionally, EDCs have been associated with altered stress responses and mood regulation (Hudecova et al. 2018; Quinnies et al. 2015), as well as various neurobehavioral disorders (de Cock et al. 2012; Kajta and Wójtowicz 2013; Miodovnik et al. 2011).

The impact of EDCs on reproductive behavior is especially well documented in fish. Numerous studies have shown that EDCs can alter courtship displays, mate choice, territorial aggression, and spawning behavior (Saaristo et al. 2019; Söffker and Tyler 2012; Tomkins et al. 2018). In addition to mating behaviors, EDC exposure can interfere with parental care, including nest building and egg fanning (Coulter et al. 2019; Saaristo et al. 2010; Bell 2005). Social dynamics, such as dominance hierarchies and territoriality, have also been shown to shift following EDC exposure, likely due to disrupted androgen or cortisol signaling (Coe et al. 2008; Filby et al. 2012). These behavioral disruptions underscore the multi‐level effects of EDCs, which may compromise not only individual reproductive success but also broader population viability.

Although fish and amphibians differ in many respects, they share important features such as external fertilization in most species, environmentally dependent reproductive cycles, and hormone‐regulated communication systems (Arcand‐Hoy and Benson 1998). Thus, findings from fish studies may offer mechanistic insights into how EDCs could interfere with comparable processes in amphibians (Kloas et al. 2009; Norris and Carr 2013). Nonetheless, amphibians possess taxon‐specific reproductive traits and behaviors, including acoustic signaling, pheromone‐based mate attraction, and sexually dimorphic skin coloration, that require direct examination (Sever and Staub 2011). Understanding how EDCs affect these specialized signaling pathways is essential for predicting the ecological consequences of pollution on amphibian populations. This is especially important because amphibians are highly sensitive to EDCs and serve as valuable, early indicators of environmental contamination (Kloas and Lutz 2006).

## 3 Materials and Methods

We extensively reviewed the studies published up to 01.01.2025 on the topic of EDCs and their impact on sexual signal production and reproductive behavior in amphibians. In the preliminary search, we included many other biological traits related to the sex and reproduction of amphibians.

We used two electronic databases for the search: Web of Science Core Collection and Google Scholar. In the database Web of Science Core Collection, the following Boolean search string was used: TS = (amphibia* OR frog* OR toad* OR newt* OR salamander* OR tadpole* OR caecilian* OR anura* OR caudata* OR urodela* OR apoda*) AND TI = (endocrine disruptor* OR endocrine* OR EDC* OR pollu* OR pharm* OR estro* OR *estra* OR andro* OR *estradiol* OR EE2* OR *gestagen* OR progesterone* OR levonorgestrel* OR diethylstilbestrol* OR aromatase inhibitor* OR flutamide* OR finasteride* OR *phenol* OR *paraben* OR *cide* OR *azine* OR methoxychlor* OR chlorpyrifos* OR organochlorine* OR *dichlorodiphenyl* OR DDD* OR DDE* OR DDT* OR azocyclotin* OR glyphosate* OR Roundup* OR butachlor* OR *phosphate* OR thiophanate* OR triadimefon* OR *azole* OR vinclozolin* OR thiophanate* OR tamoxifen* OR pyrimethanil* OR tributyltin* OR triphenyltin* OR fludioxonil* OR metaxyl* OR MAXIM OR triclosan* OR fertilizer* OR nitrate* OR ammonium* OR heavy metal* OR arsenic* OR chromium* OR cadmium* OR lead* OR surfactant* OR detergent* OR plasticizer* OR flame retardant* OR TBBPA* OR TCBPA* OR *phenyl* OR PCB* OR PBDE* OR phthalate* OR styrene* OR *chlor* OR benzene* OR UV filter* OR camphor* OR 4‐MBC* OR 3‐BC* OR trenbolone* OR microcystin* OR MCLR* OR *acid* OR salt*) AND TI = (sex* OR gonad* OR test* OR ovar* OR hormone* OR vitellogenin OR reproduct* OR fertilit* OR oocyt* OR sperm* OR breeding* OR gene* OR behavior * OR pheromone* OR scent* OR nuptial pad* OR vocal* OR acoustic* OR signal* OR call* OR communic* OR courtship* OR mate* OR color* OR phenotype*).

A similar search strategy was used in Google Scholar. The keywords for the search were chosen based on a preliminary literature review. To identify potentially relevant studies, the titles and abstracts of the retrieved articles were screened for any connection to the topic.

From this pool, studies were selected based on full‐text screening if they met the following inclusion criteria: (a) the study was experimental and involved controlled exposure of amphibians to EDCs and (b) it reported data on traits directly related to mate communication (e.g., calling behavior, pheromone production) or on traits with potential signaling function (e.g., vocal sac development, skin coloration). We focused solely on the English‐language publications, which potentially may represent a limitation of our review (Chowdhury et al. 2022; Konno et al. 2020). The journal or date of publishing the paper was not limited. Unpublished results from Master's and PhD Dissertation papers were not included. Unless stated otherwise, all studies discussed in the review were conducted under laboratory conditions using waterborne exposure and adult individuals. When mentioning changes within the reproductive system caused by EDCs, we used the nomenclature proposed by Lutz et al. (2008).

## 4 Results

**FIGURE 1:**
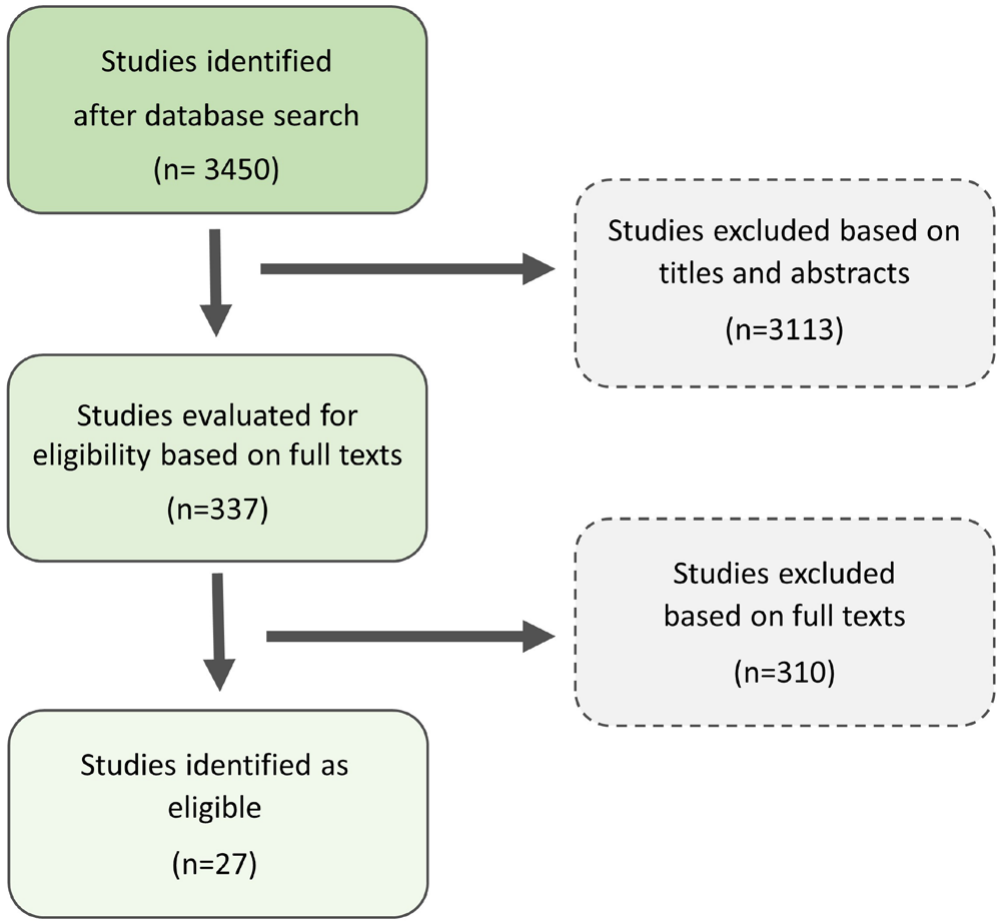
Scheme of literature search approach based on two electronic databases for the search: Web of Science Core Collection and Google Scholar.

The initial search in both Web of Science Core Collection and Google Scholar yielded over 3000 articles; the majority of which focused on EDC effects on gonadal development, hormone levels, or gene expression in amphibians. Among them, we identified only 27 eligible studies (Figure 1), from which 21 tested the effects on mating communication and behavior, and only 6 examined the impact on body coloration. This highlights how incredibly understudied this area remains, underscoring the urgent need for further research into how EDCs may influence amphibian behavior, particularly in the context of reproductive communication.

Studies, in which EDCs were found to disrupt acoustic and olfactory communication of amphibians, are summarized in Table 1, while studies on EDCs and body coloration are presented in Table 2. In the Supporting Information, a more detailed summary of all found studies, including studies in which compounds did not impact amphibians, with concentrations of EDCs calculated in different units, and additional results related to other sex‐related traits (Table S1) can be found. The names, categories, source, and environmental relevance of EDCs included in the review are presented in Table 3.

**TABLE 1:** Summary of studies and reported concentrations at which the compound affected mating behavior or related traits in amphibians.

Reference	Compound^a^	Species	Developmental stage^b^	Sex (if specified)	Time of exposure	C [mol/L]^c^	Assessed traits and parameters	The most important results
Behrends et al. (2010)	Flutamide	*Xenopus laevis*	Adult	Male	3 days	1.00E‐06	Calling activity	Decreased calling activity
Behrends et al. (2010)	Flutamide	*Xenopus laevis*	Adult	Male	3 days	1.00E‐08	Calling activity	Decreased calling activity
Efosa et al. (2017)	Diclofenac	*Xenopus laevis*	Adult	Male	8 days	9.99E‐08	Calling activity; characteristics of advertisement calls	Decreased number of advertisement calls
Hayes et al. (2010)	Atrazine	*Xenopus laevis*	Hatched tadpoles	Male	2–3 years post‐metamorphosis	1.16E‐08	Histology of larynx; morphology and histology of nuptial pads; number of successful copulations with females	Changed color of nuptial pads; changed histology of larynx; lower number of successful copulations
Hoffmann and Kloas (2010)	Vinclozolin	*Xenopus laevis*	Adult	Male	96 h	1.00E‐06	Calling activity; characteristics of advertisement calls	Decreased calling activity; changed advertisement call characteristics (call indicating less aroused males)
Hoffmann and Kloas (2010)	Vinclozolin	*Xenopus laevis*	Adult	Male	96 h	1.00E‐08	Calling activity; characteristics of advertisement calls	Decreased percentage of advertisement calls; changed call characteristics (call indicating less aroused males)
Hoffmann and Kloas (2010)	Vinclozolin	*Xenopus laevis*	Adult	Male	96 h	1.00E‐10	Calling activity; characteristics of advertisement calls	Decreased percentage of advertisement calls; changed call characteristics (call indicating less aroused males)
Hoffmann and Kloas (2012a)	17α‐ethinylestradiol	*Xenopus laevis*	Adult	Male	96 h	1.00E‐12	Calling activity	Decreased percentage of advertisement calls; changed call characteristics (call indicating less aroused males)
Hoffmann and Kloas (2012a)	17α‐ethinylestradiol	*Xenopus laevis*	Adult	Male	96 h	1.00E‐11	Calling activity	Decreased percentage of advertisement calls; changed call characteristics (call indicating less aroused males)
Hoffmann and Kloas (2012a)	17α‐ethinylestradiol	*Xenopus laevis*	Adult	Male	96 h	1.00E‐10	Calling activity	Decreased percentage of advertisement calls; changed call characteristics (call indicating less aroused males)
Hoffmann and Kloas (2012a)	17α‐ethinylestradiol	*Xenopus laevis*	Adult	Male	96 h	1.00E‐08	Calling activity; female receptivity to the call (number of females expressing phonotaxis toward male call)	Decreased percentage of advertisement calls; changed call characteristics (call indicating less aroused males); fewer females expressing phonotaxis toward male call (lower attractiveness of males)
Hoffmann and Kloas (2012a)	17α‐ethinylestradiol	*Xenopus laevis*	Adult	Male	96 h	1.00E‐06	Number and characteristics of advertisement calls	Decreased percentage of advertisement calls; changed call characteristics (call indicating less aroused males)
Hoffmann and Kloas (2012b)	17α‐ethinylestradiol	*Xenopus laevis*	Adult	Male	96 h	1.00E‐10	Calling activity; characteristics of advertisement calls	Decreased percentage of advertisement calls; changed call characteristics (call indicating less aroused males)
Hoffmann and Kloas (2012c)	Levonorgestrel	*Xenopus laevis*	Adult	Male	96 h	1.00E‐07	Calling activity; characteristics of advertisement calls	Increased percentage of advertisement calls; changed call characteristics (call indicating more sexually aroused males)
Hoffmann and Kloas (2012c)	Levonorgestrel	*Xenopus laevis*	Adult	Male	96 h	1.00E‐08	Calling activity; characteristics of advertisement calls	Increased percentage of advertisement calls; changed call characteristics (call indicating more sexually aroused males)
Hoffmann and Kloas (2012c)	Levonorgestrel	*Xenopus laevis*	Adult	Male	96 h	1.00E‐10	Calling activity; characteristics of advertisement calls	Increased percentage of advertisement calls; changed call characteristics (call indicating more aroused males)
Hoffmann and Kloas (2012d)	MDHT	Xenopus laevis	Adult		96 h	1.00E‐07	Male calling activity; characteristics of advertisement calls; female receptivity to the calls of unexposed males (number of females expressing phonotactic behaviour toward male call, latency to respond to the call, time spent near the source of male call)	Increased percentage of advertisement calls; changed call characteristics (call indicating more aroused males)
Hoffmann and Kloas (2012d)	MDHT	*Xenopus laevis*	Adult		96 h	1.00E‐05	Male calling activity; characteristics of advertisement calls; female receptivity to the calls of unexposed males (number of females expressing phonotactic behaviour toward male call, latency to respond to the call, time spent near the source of male call)	Increased percentage of advertisement calls; changed call characteristics (call indicating more aroused males); higher female receptivity to male calls (more females expressing phonotactic behaviour toward male call; quicker response to the male call)
Hoffmann and Kloas (2012d)	MDHT	*Xenopus laevis*	Adult		96 h	1.00E‐04	Male calling activity; characteristics of advertisement calls; female receptivity to the calls of unexposed males (number of females expressing phonotactic behaviour toward male call, latency to respond to the call, time spent near the source of male call)	Increased percentage of advertisement calls; changed call characteristics (call indicating more aroused males)
Hoffmann and Kloas (2016)	17α‐ethinylestradiol	*Xenopus laevis*	Adult	Male	4 days	1.00E‐10	Calling activity; characteristics of advertisement calls	Decreased percentage of advertisement calls; changed call characteristics (call indicating less aroused males)
Hoffmann and Kloas (2016)	Vinclozolin	*Xenopus laevis*	Adult	Male	4 days	1.00E‐10	Calling activity; characteristics of advertisement calls	Decreased percentage of advertisement calls; changed call characteristics (call indicating less aroused males)
Hoffmann and Kloas (2016)	p,p′DDE	*Xenopus laevis*	Adult	Male	4 days	1.00E‐09	Calling activity; characteristics of advertisement calls	Changed advertisement call characteristics (call indicating less aroused males)
Hoffmann and Kloas (2016)	p,p′DDE	*Xenopus laevis*	Adult	Male	4 days	1.00E‐11	Calling activity; characteristics of advertisement calls	Decreased percentage of advertisement calls; changed call characteristics (call indicating less aroused males)
Huang et al. (2015)	Cadmium	*Pelophylax nigromaculatus*	Adult	Male	60 days	1.00E‐06	Characteristics of advertisement calls; number of males responding with advertisement call to female call; time to first movement toward female call	Changed advertisement call characteristics (longer calls, fewer croaks); fewer males responding with advertisement call to receptive female call; longer time to first movement toward female call
Huang et al. (2015)	Cadmium	*Pelophylax nigromaculatus*	Adult	Male	60 days	1.00E‐07	Characteristics of advertisement calls; number of males responding with advertisement call to female call; time to first movement toward female call	Changed advertisement call characteristics (longer calls, fewer croaks); fewer males responding with advertisement call to receptive female call; longer time to first movement toward female call
Huang et al. (2015)	Cadmium	*Pelophylax nigromaculatus*	Adult	Male	60 days	1.00E‐08	Characteristics of advertisement calls; number of males responding with advertisement call to female call; time to first movement toward female call	Changed call characteristics (initially decreased and then increased call latency; higher call rate); longer time to first movement toward female call
Li et al. (2021)	Octylphenol	*Rana chensinensis*	Adult	Male	30 days	1.00E‐06	Mating behavior (number of pairings)	Decreased number of pairing
Park et al. (2001)	Endosulfan	*Notophthalmus viridescens*	Adult	Female	4 days	1.20E‐08	Attractiveness of females (visual selection by males; olfactory selection by males); mating success	Less males attracted toward water scented by exposed females; lower mating success; changes in pheromone gland morphology
Park et al. (2001)	Endosulfan	*Notophthalmus viridescens*	Adult	Female	4 days	2.50E‐08	Attractiveness of females (visual selection by males; olfactory selection by males); mating success	Lower mating success; changes in pheromone gland morphology
Schwendiman and Propper (2012)	17β estradiol	*Xenopus tropicalis*	Adult		30 days	1.00E‐08	Number of calls of males; number of mating behavior events: approaching mate; touching mate; arm waves; amplexus events; number of males expressing sexual behavior	Increased number of calls and arm waves in males; more males expressing sexual behavior
Schwendiman and Propper (2012)	Octylphenol	*Xenopus tropicalis*	Adult		30 days	1.00E‐07	Number of calls of males; number of mating behavior events: approaching mate; touching mate; arm waves; amplexus events; number of males expressing sexual behavior	Increased number of calls and arm waves in males; more males expressing sexual behavior
Schwendiman and Propper (2012)	Octylphenol	*Xenopus tropicalis*	Adult		30 days	1.00E‐08	Number of calls of males; number of mating behavior events: approaching mate; touching mate; arm waves; amplexus events; number of males expressing sexual behavior	Increased number of calls in males; more males expressing sexual behavior
Secondi et al. (2009)	Sodium nitrate	*Lissotriton helveticus*	Adult	Male	10 days	0.882	Secondary sexual characteristics (tail height; hind‐foot web area); attractiveness of males (visual selection by females; olfactory selection by females)	Fewer females attracted to water scented by exposed males
Secondi et al. (2013)	Sodium nitrate	*Lissotriton helveticus*	Adult	Male	10 days	0.882	Courtship behavior (occurrence of courtship behavior; number and duration of fanning; number of spermatophore deposition and transfer)	More males expressing courtship behavior
Zhang et al. (2022)	NAs	*Xenopus tropicalis*	Adult	Male	5 days	20 mg/L^d^	Calling activity; characteristics of advertisement calls	Decreased calling activity

^a^ MDHT, methyldihydrotestosterone; NAs, naphthenic acids; p,p′DDE, dichlorodiphenyldichloroethylene.

^b^ Gosner stage – developmental stage according to Gosner (1960); NF stage – developmental stage according to Nieuwkoop and Faber (2020).

^c^ Calculated assuming dilute aqueous solution of density equal to the density of water in normal conditions (997 kg/m^3^).

^d^ Can't calculate concentration/provide molar mass, due to substance tested being a mixture of different compounds.

**TABLE 2:** Summary of studies and concentrations at which the compound affected body coloration of amphibians.

Reference	Compound^a^	Species	Developmental stage^b^	Time of exposure	C [mol/L]^c^	Assessed traits and parameters	The most important results
Greenberg (1942)	Testosterone	*Acris gryllus*	Juvenile	3 weeks	implants under the skin, unspecified dosage	Sexually dimorphic body coloration, presence of vocal sacs	Coloration characteristic for adult females in males and females
Greenberg (1942)	Testosterone	*Acris gryllus*	Juvenile	5 weeks	implants under the skin, unspecified dosage	Sexually dimorphic body coloration, presence of vocal sacs	Coloration characteristic of adult females in males and females
Greenberg (1942)	Testosterone	*Acris gryllus*	Juvenile	3 months	implants under the skin, unspecified dosage	Sexually dimorphic body coloration, presence of vocal sacs	Coloration characteristic of adult females in males and females
Hayes and Menendez (1999)	17β estradiol	*Hyperolius argus*	Gosner stage 21	Up to Gosner stage 46	3.67E‐05	Body coloration; presence of vocal sacs	Coloration characteristic for adult females in 100% of individuals; no vocal sacs
Hayes and Menendez (1999)	17β estradiol	*Hyperolius argus*	Gosner stage 21	Up to Gosner stage 46	3.67E‐04	Body coloration; presence of vocal sacs	Coloration characteristic for adult females in 100% of individuals; no vocal sacs
Noriega and Hayes (2000)	17β estradiol	*Hyperolius argus*	Within 24 h of forelimb emergence	20 days	2.80E‐07	Sexually dimorphic body coloration	Premature body coloration in females
Noriega and Hayes (2000)	17β estradiol	*Hyperolius argus*	Within 24 h of forelimb emergence	20 days	3.67E‐07	Sexually dimorphic body coloration	Premature body coloration in females
Noriega and Hayes (2000)	17β estradiol	*Hyperolius argus*	Within 24 h of forelimb emergence	20 days	3.67E‐06	Sexually dimorphic body coloration	Premature body coloration in females
Noriega and Hayes (2000)	o,p′DDT	*Hyperolius argus*	Within 24 h of forelimb emergence	20 days	2.80E‐07	Sexually dimorphic body coloration	Premature body coloration in females
Noriega and Hayes (2000)	o,p′DDT	*Hyperolius argus*	Within 24 h of forelimb emergence	20 days	2.80E‐06	Sexually dimorphic body coloration	Premature body coloration in females
Noriega and Hayes (2000)	o,p′DDE	*Hyperolius argus*	Within 24 h of forelimb emergence	20 days	2.80E‐06	Sexually dimorphic body coloration	Premature body coloration in females
Noriega and Hayes (2000)	o,p′DDD	*Hyperolius argus*	Within 24 h of forelimb emergence	20 days	2.80E‐06	Sexually dimorphic body coloration	Premature body coloration in females
Richards (1982)	Testosterone	*Hyperolius viridiflavus*	From the start of feeding	Up to the emergence of forelimbs; 1‐min bath per day	1.73E‐04	Sexually dimorphic body coloration	Coloration characteristic for adult females in males and females
Richards (1982)	17β estradiol	*Hyperolius viridiflavus*	From the start of feeding	Up to emergence of forelimbs; 1‐min bath per day	1.83E‐07	Sexually dimorphic body coloration	Coloration characteristic of adult females in males and females
Richards (1982)	17β estradiol	*Hyperolius viridiflavus*	From the start of feeding	Up to the emergence of forelimbs; 1‐min bath per day	3.66E‐07	Sexually dimorphic body coloration	Coloration characteristic of adult females in males and females
Richards (1982)	17β estradiol	*Hyperolius viridiflavus*	From the start of feeding	Up to emergence of forelimbs; 1‐min bath per day	1.83E‐06	Sexually dimorphic body coloration	Coloration characteristic of adult females in males and females
Richards (1982)	17β estradiol	*Hyperolius viridiflavus*	From the start of feeding	Up to the emergence of forelimbs; 1‐min bath per day	3.66E‐06	Sexually dimorphic body coloration	Coloration characteristic of adult females in males and females
Scaia et al. (2019)	Nonylphenol	*Lithobates catesbeianus*	Gosner stage 32‐35	14 days	4.54E‐06	Body coloration	Darker body pigmentation
Scaia et al. (2019)	Nonylphenol	*Lithobates catesbeianus*	Gosner stage 32‐35	14 days	4.54E‐05	Body coloration	Darker body pigmentation
Scaia et al. (2019)	Nonylphenol	*Lithobates catesbeianus*	Gosner stage 32‐35	14 days	4.54E‐04	Body coloration	Darker body pigmentation
Ujhegyi and Bókony (2020)	Glyphogan Classic	*Bufo bufo*	Gosner stage 25	Up to Gosner stage 42	1.31E‐05	Sexually dimorphic body coloration	3 mixed‐sex individuals with changes in body coloration

^a^ DDD, dichlorodiphenyldichloroethane; DDE, dichlorodiphenyldichloroethylene; DDT, dichlorodiphenyltrichloroethane.

^b^ Gosner stage – developmental stage according to Gosner (1960); NF stage – developmental stage according to Nieuwkoop and Faber (2020).

^c^ Calculated assuming dilute aqueous solution of density equal to the density of water in normal conditions (997 kg/m^3^).

**TABLE 3:** Summary of the endocrine‐disrupting compounds included in the review.

Common name	Group of compounds	Description and relevance	Reference
17α‐ethinylestradiol (EE2)	Estrogens	Synthetic estrogen used in contraceptive and replacement therapy drugs, commonly found in the environment	Wise et al. (2010)
17β‐estradiol (E2)	Estrogens	Natural hormone excreted by animals, present also in therapeutic drugs, commonly found in the environment	Wise et al. (2010)
Ammonium nitrate	Fertilizers	Ingredient of fertilizers, intensively used in agriculture, commonly found in the environment	Daam et al. (2020)
Atrazine (ATZ)	Herbicides	Herbicide intensively used in agriculture, commonly found in the environment	Rohr and McCoy (2010)
Cadmium (Cd)	Heavy metals	Used in e.g., Pesticides, nickel‐cadmium batteries, color pigments, the wood industry, commonly found in the environment	Järup and Åkesson (2009)
Dichlorodiphenyldichloroethane (DDD)	Insecticides	Breakdown product of DDT, highly persistent and commonly found in the environment	Agency for Toxic Substances and Disease Registry (2024)
Dichlorodiphenyldichloroethylene (DDE)	Insecticides	Breakdown product of DDT, highly persistent and commonly found in the environment	Agency for Toxic Substances and Disease Registry (2024)
Dichlorodiphenyltrichloroethane (DDT)	Insecticides	Insecticide used in large quantities in agriculture and vector control in the past, highly persistent and commonly found in the environment	Agency for Toxic Substances and Disease Registry (2024)
Diclofenac (DCF)	Pharmaceuticals	Widely used anti‐inflammatory drug, commonly found in the environment	Efosa et al. (2017)
Endosulfan	Insecticides	Insecticide intensively used in agriculture, commonly found in the environment	Silva and Gammon (2009)
Flutamide (FLU)	Antiandrogens	Therapeutic drug with little environmental relevance, sporadically can occur in wastewater	Behrends et al. (2010)
Fulvestrant	Antiestrogens	Therapeutic drug with little environmental relevance, sporadically can occur in wastewater	Parrella et al. (2014)
Levonorgestrel (LNG)	Gestagens	Synthetic analog of progesterone, used in contraceptive drugs, hormonal replacement and cancer therapies, commonly found in the environment	Oropesa and Guimarães (2021)
Methyldihydrotestosterone (MDHT)	Androgens	Synthetic androgen currently withdrawn from the medical application, used as a model androgen in studies on EDCs, with little environmental relevance alone	Hoffmann and Kloas (2012a, 2012b, 2012c, 2012d)
Naphthenic acid fraction compounds (nafcs)	Petroleum ingredients	Carboxylic acids found in crude oils, fuel additives, emulsifiers, surfactants, enter freshwater ecosystems mainly after oil spills	Robinson et al. (2023)
Naphthenic acids (nas)	Petroleum ingredients	Carboxylic acids found in crude oils, fuel additives, emulsifiers, surfactants, enter freshwater ecosystems mainly after oil spills	Zhang et al. (2022)
Nonylphenol (NP)	Phenols	Compound widely used in surfactants and detergents, commonly found in the environment	Mosconi et al. (2002)
Octylphenol (OP)	Phenols	Compound widely used in surfactants and detergents, commonly found in the environment	Mosconi et al. (2002)
Progesterone (P)	Gestagens	Natural gestagen used in human medicine and veterinary drugs, commonly found in the environment	Lange et al. (2002)
Sodium nitrate	Fertilizers	Ingredient of fertilizers, intensively used in agriculture, commonly found in the environment	Poulsen et al. (2018)
Tamoxifen (TAM)	Antiestrogens	Therapeutic drug with moderate environmental relevance, sporadically can occur in wastewater	Orias et al. (2015)
Testosterone (T)	Androgens	Natural hormone excreted by animals, present also in therapeutic drugs, commonly found in the environment	Thomas et al. (2002)
Vinclozolin (VIN)	Fungicides	Fungicide intensively used in agriculture, commonly found in the environment	Feijó et al. (2021)

## 5 Impact on Behavior by Disruption of Acoustic Communication

Hormones play a key role in the development and maintenance of secondary sexual characteristics and reproductive behaviors in amphibians, including sexual communication, partner receptivity, and mating (for reviews, see Sever and Staub 2011; Woodley 2011). These hormonally mediated traits may be susceptible to disruption by the presence of EDCs in the environment. In the majority of anuran species, acoustic communication is the primary mechanism used to locate and attract a mate and initiate reproduction (Colafrancesco and Gridi‐Papp 2016; Wake and Koo 2018; Wells and Schwartz 2006). Research indicates that both the production and perception of acoustic signals in amphibians are modulated by hormones such as androgens, estrogens, gonadotropin‐releasing hormone, and neuropeptides, including arginine vasotocin (for reviews, see Arch and Narins 2009; Woodley 2011). As a result, EDCs that interfere with these hormonal pathways may disrupt acoustic communication, ultimately impairing mate selection and breeding success.

The well‐established model for studying the effects of EDCs on vocal signals in amphibians is the African clawed frog *Xenopus laevis* (Hall and Kelley 2021). Males of this species produce a variety of vocalizations, including ticking, rasping, croaking, and growling. Advertisement calls, which signal readiness to mate, consist primarily of ticking and croaking. Growling—a low‐frequency, continuous vibration—is typically used to deter rival males, while rasping is thought to be associated with aggressive interactions or a reduced level of sexual arousal (Leininger and Kelley 2015).

Studies on *X. laevis* have demonstrated that androgens are critical for the development of the male vocal apparatus, including the larynx, laryngeal muscles, and motoneurons. The size, structure, and innervation of the larynx, as well as the neural circuits responsible for call production in the brain, are sexually dimorphic in this species (Hall and Kelley 2021; Zornik and Yamaguchi 2008). It has been shown that EDCs can interfere with the normal development of this complex system when exposure occurs during early life stages, thereby disrupting acoustic communication (Carr et al. 2003). Notably, EDCs may also affect vocal signals in adult individuals. This is likely due to the fact that adult amphibians retain a certain degree of plasticity within their vocal system. For example, ovariectomized females of *X. laevis* exposed to testosterone (T) via implants for 13 weeks began producing advertisement calls typical for males. This modification of the call was linked to changes within the vocal apparatus (Potter et al. 2005).

### 5.1 Disruption of Vocal Signals by Endocrine Disruptors: Estrogenic and Anti‐Androgenic Compounds

Evidence confirming that estrogenic and anti‐androgenic compounds commonly found in the environment can alter amphibian vocal behavior is provided by 10 studies (see Tables 1 and 3). In one of them, Behrends et al. (2010) demonstrated that brief waterborne exposure to the anti‐androgen flutamide at a low concentration (1 × 10^−8^ M) significantly suppressed the mating calls of adult male *Xenopus laevis* . Similarly, exposure to the non‐steroidal anti‐inflammatory drug diclofenac (DCF)—which is known to interfere with estrogenic pathways—at an environmentally relevant concentration (1 × 10^−7^ M) resulted in a notable reduction of the vocal activity of *X. laevis* males (Efosa et al. 2017). Given that vocalizations in many anuran species play a key role in attracting mates, a reduction in call output could significantly impair male reproductive success in natural settings. However, this hypothesis has not yet been directly tested in the studies cited (Behrends et al. 2010; Efosa et al. 2017), and the ecological consequences of reduced vocal activity remain largely speculative.

It is also important to note that experimental studies investigating the effects of EDCs on amphibian vocalizations often involve methodological constraints that may influence outcomes. In all reviewed studies, males were pharmacologically stimulated with human chorionic gonadotropin (hCG) prior to chemical exposure (see Table S1). This technique is used to trigger vocal behavior in laboratory conditions, particularly outside the breeding season (Schmidt 1966). However, the use of hCG may not accurately reflect natural physiological states and could affect the animal's calling patterns or hormone sensitivity (Efosa et al. 2017).

This concern was illustrated in the study by Efosa et al. (2017), where male frogs exposed to diclofenac (DCF) without hCG pre‐treatment showed a somewhat different vocal response compared to hCG‐stimulated males. They expressed not only lower vocal output but also vocalized a higher proportion of rasping, which can be interpreted as an additional signal of low sexual arousal (Efosa et al. 2017). These findings suggest that hormonal induction protocols may interact with tested compounds, potentially confounding experimental results. Therefore, when possible, stimulating animals to vocal activity by additional substances should be avoided to capture a more realistic view of EDCs' action.

In other experimental studies, the calling activity of adult *Xenopus laevis* males was reduced following exposure to agrochemicals known for their anti‐androgenic action, including the insecticide dichlorodiphenyldichloroethylene (DDE) (Hoffmann and Kloas 2016) and the fungicide vinclozolin (VIN) (Hoffmann and Kloas 2010, 2016). Males exposed to relatively low and environmentally relevant concentrations of DDE (1 × 10^−11^ M) and VIN (1 × 10^−10^ M) produced significantly fewer advertisement calls; in the case of VIN, they exhibited a notable increase in the proportion of ticking sounds (Hoffmann and Kloas 2016). Furthermore, the advertisement calls of VIN‐exposed males were characterized by longer pauses between clicks and a lower click rate. This pattern of calling, especially when combined with a reduced number of signals, may decrease the male's chances of attracting a female.

Importantly, DDE had a slightly different impact on males depending on the concentration (Hoffmann and Kloas 2016). Males exposed to 1 × 10^−11^ M DDE produced more growling sounds (deterring other males), while in the higher concentration (1 × 10^−9^ M) more ticking sounds (expressing higher sexual arousal). It remains unclear how such modifications may impact the chances of males to successfully reproduce.

The synthetic estrogenic compound 17α‐ethinylestradiol (EE2) has also been shown to significantly affect the calling behavior of *X. laevis* males, at environmentally relevant concentrations of 1 × 10^−10^ M (Hoffmann and Kloas 2012b, 2016) and 1 × 10^−12^ M (Hoffmann and Kloas 2012a). As expected for an estrogenic compound, exposed males produced fewer advertisement calls (Hoffmann and Kloas 2016). In addition, the composition of the calls was altered. Males produced a higher amount of rasping and growling than the respective control animals, reflecting a lower sexual arousal state (Hoffmann and Kloas 2012a, 2012b, 2016). Both the spectral (Hoffmann and Kloas 2012a, 2012b, 2016) and temporal parameters (Hoffmann and Kloas 2012a, 2012b) of advertisement calls were affected, indicating a shift in signal structure. As a result, fewer females moved toward the modified calls of males (Hoffmann and Kloas 2012a).

Importantly, the effect of EE2 on male calls disappeared 6 weeks after exposure, indicating that the impact of the compound was temporary (Hoffmann and Kloas 2012a). Interestingly, the effect of EE2 was also reduced when males were simultaneously exposed to anti‐estrogenic compounds, such as tamoxifen or fulvestrant, which can suppress the action of synthetic estrogens (Hoffmann and Kloas 2012c). These findings highlight the importance of studying the combined effects of EDCs, especially those that amphibians are likely to encounter together in natural environments.

The natural estrogen 17β‐estradiol (E2) and octylphenol (OP), a compound with estrogenic properties, have also been found to significantly affect the acoustic communication of amphibians. Interestingly, in a study using another model species—the Western clawed frog ( *Xenopus tropicalis* )—these estrogenic compounds had a stimulating, rather than suppressing, effect on males. Males exposed to E2 and OP at a concentration of 1 × 10^−8^ M produced a higher number of advertisement calls. Additionally, males exposed to OP at 1 × 10^−7^ M showed increased arm waving, a behavior potentially linked to pheromone release. These changes could be interpreted as a sign of increased sexual arousal. In contrast, female behavior remained unchanged following exposure to either E2 or OP (Schwendiman and Propper 2012).

These unexpected results highlight the importance of comparing the effects of different EDCs—even within the same chemical group. For example, could the natural estrogen E2 have different effects than the synthetic compound EE2 used in previous studies? Similarly, do males of *X. tropicalis* respond differently to estrogenic compounds compared to *X. laevis* ? These questions underline the need for species‐specific research and a more nuanced understanding of how EDCs influence amphibian behavior.

Disruption of mating calls in adult *X. tropicalis* males was also observed following exposure to naphthenic acids (NAs)—petroleum‐derived compounds suspected to have weak estrogenic and anti‐androgenic properties—at a concentration of 20 mg/L (Zhang et al. 2022). Males exposed to this environmentally relevant concentration showed reduced vocal output and shorter calling duration, which may reflect lower sexual arousal. Interestingly, the effect of NAs was reversible: after 2 weeks of recovery in clean water, the calling activity of males returned to normal (Zhang et al. 2022).

In contrast, exposure of the wood frog ( *Lithobates sylvaticus* ) to naphthenic acid fraction compounds (NAFCs)—chemically related substances that may also have anti‐androgenic properties—at concentrations of 5 or 10 mg/L did not alter mating behavior (Robinson et al. 2023). This difference may be due to chemical variation between NAs and NAFCs or species‐specific sensitivity to these compounds. These findings reinforce the importance of conducting comparative studies across multiple amphibian taxa to better understand the diverse effects of EDCs and to identify species that may be particularly vulnerable to environmental contamination (Frątczak et al. 2025).

### 5.2 Disruption of Vocal Signals by Endocrine Disruptors: Other Compounds

To date, only one study has investigated the effects of a typical androgen on the vocal activity of amphibians (Hoffmann and Kloas 2012d). It can be hypothesized that androgens and anti‐estrogenic compounds may have a stimulating effect on male mating behavior. An experiment on adult male *Xenopus laevis* showed that exposure to the androgenic compound methyldihydrotestosterone (MDHT) at concentrations as low as 1 × 10^−7^ M altered their advertisement calls, with males producing fewer rasping elements—vocalizations usually linked to a less sexually aroused state—suggesting enhanced sexual arousal. Interestingly, MDHT also affected female behavior: exposure to a concentration of 1 × 10^−5^ M increased female receptivity to normal male advertisement calls played through speakers (Hoffmann and Kloas 2012d).

Another understudied group of EDCs in the context of amphibian behavior are gestagens (Hoffmann and Kloas 2012c; Säfholm et al. 2014). Notably, interesting results were observed after exposing adult *X. laevis* males to levonorgestrel (LNG)—a synthetic progestin—at a concentration as low as 1 × 10^−7^ M. Exposed males produced altered advertisement calls, with a higher number of calls and a lower proportion of rasping, indicating a more sexually aroused state. It can be hypothesized that such call patterns may increase the likelihood of attracting a mate (Hoffmann and Kloas 2012c).

Interestingly, the natural analog progesterone did not affect male calling parameters in the same way. This difference may be due to the mildly androgenic activity of LNG (Hoffmann and Kloas 2012c). These findings underscore that the effects of synthetic analogs can differ significantly from those of natural hormones. This highlights the importance of carefully selecting compounds for experimental studies, ensuring that they reflect the types of substances amphibians are likely to encounter in their environment. Furthermore, the choice of positive controls requires special attention, as natural hormones may act differently than their synthetic counterparts.

Heavy metals, such as cadmium (Cd), can also significantly affect the vocal activity of amphibians (Huang et al. 2015). These effects are believed to result either from weak interactions with estrogenic pathways or through neurotoxic mechanisms (Huang et al. 2015; Järup and Åkesson 2009). This was demonstrated in the dark‐spotted frog ( *Pelophylax nigromaculatus* ), a species in which males produce advertisement calls during the breeding season to attract females (Cheong et al. 2013). According to Huang et al. (2015), receptive females respond vocally, prompting males to emit specific reply calls and approach the source of the sound. In their long‐term study, exposure to cadmium (Cd) altered male responses to female calls. Males exposed to higher Cd concentrations (1 × 10^−6^ and 1 × 10^−7^ M) produced longer calls with fewer croaks, while at lower concentrations (1 × 10^−8^ M), call latency initially decreased and then increased, and an elevated call rate was observed. Additionally, exposed males were less likely to approach female calls, and a significant proportion did not vocalize at all.

It was confirmed in a later study that low doses of cadmium cause histological changes in the larynx of both male and female *P. nigromaculatus* (Duan and Huang 2016). These structural alterations may explain the changes in vocal signaling and, importantly, suggest that the effects could be permanent.

## 6 Impact on Behavior by Disruption of Olfactory Communication

In amphibian species that do not rely on vocalizations for mate attraction, pheromones serve as key signals for communication between individuals (Kikuyama et al. 2002). Although chemical communication in amphibians has been studied for several decades, particularly in Caudata, many aspects remain poorly understood (Woodley 2015). Foundational work in salamanders identified the presence of courtship pheromones produced by mental glands (Houck and Reagan 1990; Kikuyama et al. 2002), and more recent studies have characterized specific pheromones and their behavioral effects (Rollmann et al. 1999; Woodley 2015). However, the chemical identity, variation, and perception of these signals remain underexplored across most amphibian taxa, especially in Anura (Woodley 2015).

Available data indicate that pheromone production and reception in amphibians are regulated by prolactin and steroid hormones (Kikuyama et al. 2002; Sever and Staub 2011). As a result, EDCs present in the environment may alter these hormonal pathways, thereby affecting both the production and perception of pheromones.

For example, the insecticide endosulfan, suspected of anti‐androgenic and weakly estrogenic activity (Silva and Gammon 2009), has been shown to disrupt pheromone signaling in the red‐spotted newt ( *Notophthalmus viridescens* ). Female newts exposed to endosulfan at a concentration as low as 1.2 × 10^−8^ M exhibited changes in their pheromonal glands, and their scent became less attractive to males (Park et al. 2001).

Another compound, the fertilizer sodium nitrate, affected the courtship behavior of the palmate newt ( *Lissotriton helveticus* ) at a concentration of 8.82 × 10^−4^ M. Exposed males showed a higher probability of displaying courtship behavior, which may indicate that sodium nitrate disrupts pheromone production, prompting males to compensate with increased visual displays (Secondi et al. 2013). Supporting this interpretation, *L. helveticus* females were less attracted to the scent of males exposed to the same concentration of sodium nitrate in another study (Secondi et al. 2009).

In contrast, ammonium nitrate, another common fertilizer ingredient, had no significant effect on the courtship behavior of the Iberian newt ( *Lissotriton boscai* ) under similar experimental conditions (Ortiz‐Santaliestra et al. 2009). The mechanisms of endocrine disruption for both sodium and ammonium nitrates remain poorly understood, and their effects do not align clearly with classical androgenic or estrogenic pathways (Poulsen et al. 2018; Daam et al. 2020).

## 7 Potential Effects of Endocrine Disruptors via Disruption of Physical Traits

EDCs can reduce amphibian reproductive success not only by disrupting mating behaviors directly but also by altering hormone‐dependent traits involved in communication between mates. Several such traits mentioned throughout this review—such as the presence of vocal sacs (Hayes and Menendez 1999), laryngeal structure (Huang et al. 2015; Duan and Huang 2016), and nuptial pad morphology (Hayes et al. 2010)—are strongly hormone‐dependent and play a critical role in producing or receiving mating signals. Altered secondary sexual traits, such as tail shape and foot webbing in *Lissotriton helveticus* , may also significantly influence female mate choice (Secondi et al. 2009, 2013).

Another hormone‐regulated trait with a signaling function that may be disrupted by EDCs is skin coloration. It is a hormone‐regulated trait that plays an important role in sexual communication in many amphibian species (Sever and Staub 2011). In anurans, for example, sexually dimorphic coloration often develops during the breeding season and may serve as a visual cue for mate recognition, assessment of sexual maturity, or indication of reproductive readiness (Rojas et al. 2023). The presence of sex steroid hormone receptors in the skin of both larvae and adults (Chieffi et al. 1975; Sever and Staub 2011) supports its role as a hormone‐responsive signaling trait. Additionally, the genetic basis of coloration has been linked to sex chromosomes in some species, such as *Rana rugosa* , where a sex‐linked color gene has been identified (Miura et al. 2011).

Given this background, skin coloration may be susceptible to disruption by EDCs, potentially altering communication between mates (Ujhegyi and Bókony 2020). However, to date, this hypothesis has not been experimentally tested. As such, we present data on substances that affect coloration as a case study of a physiological trait with potential indirect consequences for sexual communication, and as an area that needs further investigation due to its possible significance for amphibian reproductive success.

### 7.1 Disruption of Sexually Dimorphic Body Coloration

One of the best‐documented examples of EDC‐induced disruption of sexually dimorphic coloration comes from studies on reed frogs. In the Argus reed frog ( *Hyperolius argus* ), body coloration is a sexually dimorphic trait that plays an important role in mate recognition (Rojas et al. 2023). The organochlorine insecticide dichlorodiphenyltrichloroethane (DDT) and its congeners, known for their anti‐androgenic activity (Agency for Toxic Substances and Disease Registry 2024), have been found to influence body coloration in this species. Females exposed to o,p′‐DDT at concentrations of 2.8 × 10^−7^ M and 2.8 × 10^−6^ M, as well as to o,p′‐DDD and o,p′‐DDE at 2.8 × 10^−6^ M, prematurely developed adult‐type skin coloration. In contrast, exposure to positional isomers of these compounds, p,p′‐DDT, p,p′‐DDE, and p,p′‐DDD did not result in observable changes in coloration (Noriega and Hayes 2000).

In another study, *H. argus* males exposed to estradiol (E2) at relatively high concentrations (3.65 × 10^−4^ M and 3.65 × 10^−5^ M) during the larval stage developed dorsal coloration typical of females. These males also failed to develop vocal sacs. In contrast, exposure to testosterone (T) led to the development of the male phenotype, including male‐typical coloration and development of vocal sacs, in both genetic males and females (Hayes and Menendez 1999).

Interestingly, in the common reed frog ( *Hyperolius viridiflavus* ), exposure to both E2 (1.83 × 10^−7^ M) or T (1.73 × 10^−4^ M) during larval and juvenile stages caused the development of a phenotype typical for adult females: bright green or yellow body coloration, in genetic males and females. Importantly, this effect was reversible; after a few weeks without further exposure, individuals returned to their normal coloration (Richards 1982).

Similar effects of hormone manipulation or EDC exposure on body coloration have also been observed in other amphibian species, further supporting the idea that skin pigmentation is hormonally regulated and susceptible to disruption. In *Acris gryllus* (cricket frog), testosterone implants (dose not precisely reported) caused both juvenile males and females to develop male‐typical breeding coloration, including yellow and dark throat pigmentation (Greenberg 1942).

In the common toad ( *Bufo bufo* ), dorsal coloration is moderately sexually dimorphic, with males typically showing brighter, more yellow‐green skin, and females appearing darker and more reddish. Larval exposure to the synthetic estrogen 17α‐ethinylestradiol (EE2) or the estrogenic herbicide glyphosate led to the appearance of mixed‐sex individuals with duller, reddish‐brown coloration, distinct from that of both normal males and females. Treatment with 1 μg/L EE2 resulted in 100% female phenotypes with typical female coloration, while mixed‐sex individuals occurred at 1 ng/L EE2 and 3 μg/L glyphosate. However, it should be noted that the number of mixed‐sex individuals observed was very small (Ujhegyi and Bókony 2020).

Additionally, in the American bullfrog ( *Lithobates catesbeianus* ), short‐term exposure (14 days) of females to nonylphenol (NP) resulted in darkening of body pigmentation (Scaia et al. 2019). Worth noting, effects on skin coloration of amphibians may be useful as a potential biomarker of EDC exposure in wild populations (Orton et al. 2023).

## 8 Summary and Future Directions

EDCs can significantly impact the mating behavior of amphibians. As we show in our review, endocrine disruptors interfere with acoustic, olfactory, and potentially visual communication of amphibians, which are essential for mate selection and further stages of courtship and breeding. Such disruptions may negatively impact not only the reproductive success of individuals but also the dynamics of entire populations and ultimately the survival of amphibian species. Notably, even relatively short exposure periods induce significant changes, highlighting this as an important direction for future research.

There are still significant research gaps in our understanding of the impact of EDCs on amphibian mating behavior. First of all, many studies test only the immediate impact of EDCs on the behavior of amphibians (e.g., Behrends et al. 2010; Hoffmann and Kloas 2012a, 2012b, 2012c, 2012d; Hoskins et al. 2017; Park et al. 2001, for more examples see Table S1). There is a strong need for more research on the long‐term and multi‐generational consequences of chronic exposure to EDCs on the mating behavior and reproduction of amphibians.

Moreover, most studies on EDCs and amphibian behavior focused only on the effects of single chemicals and no additional stressors (See Table S1). The exception is the study of Hoffmann and Kloas (2012b), in which the effect of EE2 was tested in combination with anti‐estrogenic compounds. It should be taken into account that in the natural environment, amphibians have contact with many different substances of natural (Iglesias‐Carrasco et al. 2017) and anthropogenic origin (Swanson et al. 2018), as well as other stressors, such as competition with other mates (Uzendoski et al. 1993), presence of pathogens (Paetow et al. 2023), parasites (DuRant et al. 2015), and predators (LaFiandra et al. 2008; Uzendoski et al. 1993). These stressors can have synergistic or antagonistic actions that cannot be observed by studying a single contaminant in the simplified experimental protocols (Awkerman et al. 2024).

As we highlighted earlier, studies on EDCs need to encompass various substances, even those that are theoretically similar, such as natural and synthetic counterparts. Even if their chemical structures are similar, it seems that they can exhibit significant differences in their impact on amphibians (Hoffmann and Kloas, 2012). Additionally, synthetic substances may differ from natural ones in terms of environmental stability (Jürgens et al. 2002), which can affect their availability and bioactivity in organisms. We need studies on a wide range of compounds to better understand the potential risks associated with exposure to EDCs and enable more accurate predictions of their impact on organisms in natural environments.

Bias in the use of the model species, mainly *X. laevis* , is a universal problem in all studies encompassing the impact of pollutants on amphibians (Frątczak et al. 2025; Schiesari et al. 2007). Species of amphibians may have different sensitivities to EDCs (Rozenblut‐Kościsty et al. 2019; Tamschick, Rozenblut‐Kościsty, Ogielska, Kekenj, et al. 2016; Tamschick, Rozenblut‐Kościsty, Ogielska, Lehmann, et al. 2016), as well as varying degrees of risk of exposure to EDCs in the environment due to their ecology. For example, while *X. laevis* (Zhang et al. 2022) is a fully aquatic species, *L. sylvaticus* (Robinson et al. 2023) is a terrestrial species with a short, explosive mating season (AmphibiaWeb, n.d.). Furthermore, *X. laevis*, used in the majority of EDC studies, employs a unique underwater vocalization mechanism distinct from the air‐driven calls typical of terrestrial Neobatrachian species (Walkowiak 2006). These ecological and mechanistic differences highlight the need for studies to include a broader range of amphibian taxa to better understand the diverse impacts of EDCs on communication across species.

Taken together, existing evidence demonstrates that EDCs pose a serious but still underexplored threat to amphibian reproductive communication. To fully understand their ecological impact, future research must move beyond simplified models and experimental conditions. Only then can we accurately assess the risks EDCs pose to amphibian biodiversity and reproductive success in the wild.

## Author Contributions

**Martyna Frątczak:** conceptualization (equal), investigation (lead), visualization (lead), writing – original draft (lead), writing – review and editing (equal). **Mikołaj Kaczmarski:** conceptualization (equal), writing – review and editing (equal). **Katarzyna Szkudelska:** supervision (equal), writing – review and editing (equal). **Piotr Tryjanowski:** conceptualization (equal), methodology (lead), supervision (equal), writing – review and editing (equal).

## Conflicts of Interest

The authors declare no conflicts of interest.

## Supporting information


**Table S1:** Summary of the studies on the impact of endocrine‐disrupting compounds on mating behavior, sexual communication and body coloration of amphibians, including the type of compound, species studied, developmental stage, time of exposure and the most significant results.

## Data Availability

All data used in the review are available in the Supporting Information.

## References

[ece371986-bib-0001] Agency for Toxic Substances and Disease Registry . 2024. Toxicological Profile for DDT, DDE, and DDD. U.S. Department of Health and Human Services, Public Health Service. https://www.atsdr.cdc.gov/toxprofiles/tp.asp?id=81&tid=20. 37023235

[ece371986-bib-0003] AmphibiaWeb . n.d. Accessed January 30, 2024. https://amphibiaweb.org/.

[ece371986-bib-0004] Arcand‐Hoy, L. D. , and W. H. Benson . 1998. “Fish Reproduction: An Ecologically Relevant Indicator of Endocrine Disruption.” Environmental Toxicology and Chemistry 17: 49–57. 10.1002/etc.5620170108.

[ece371986-bib-0005] Arch, V. S. , and P. M. Narins . 2009. “Sexual Hearing: The Influence of Sex Hormones on Acustic Communication in Frogs.” Hearing Research 252: 15–20. 10.1016/J.HEARES.2009.01.001. 19272318 PMC2722832

[ece371986-bib-0006] Awkerman, J. A. , D. A. Glinski , W. M. Henderson , R. V. Meter , and S. T. Purucker . 2024. “Framework for Multi‐Stressor Physiological Response Evaluation in Amphibian Risk Assessment and Conservation.” Frontiers in Ecology and Evolution 12: 1–16. 10.3389/fevo.2024.1336747. PMC11636185 39679000

[ece371986-bib-0007] Baker, N. J. , B. A. Bancroft , and T. S. Garcia . 2013. “A Meta‐Analysis of the Effects of Pesticides and Fertilizers on Survival and Growth of Amphibians.” Science of the Total Environment 449: 150–156. 10.1016/j.scitotenv.2013.01.056. 23422494

[ece371986-bib-0008] Băncilă, R. I. , M. Lattuada , and N. Sillero . 2023. “Distribution of Amphibians and Reptiles in Agricultural Landscape Across Europe.” Landscape Ecology 38: 861–874. 10.1007/s10980-022-01583-w.

[ece371986-bib-0009] Behrends, T. , R. Urbatzka , S. Krackow , A. Elepfandt , and W. Kloas . 2010. “Mate Calling Behavior of Male South African Clawed Frogs ( *Xenopus laevis* ) is Suppressed by the Antiandrogenic Endocrine Disrupting Compound Flutamide.” General and Comparative Endocrinology 168: 269–274. 10.1016/j.ygcen.2010.01.017. 20138181

[ece371986-bib-0010] Bell, A. M. 2005. “Behavior al Differences Between Individuals and Two Populations of Stickleback ( *Gasterosteus aculeatus* ).” Journal of Evolutionary Biology 18: 464–473. 10.1111/j.1420-9101.2004.00817.x. 15715852

[ece371986-bib-0011] Bókony, V. , B. Üveges , N. Ujhegyi , et al. 2018. “Endocrine Disruptors in Breeding Ponds and Reproductive Health of Toads in Agricultural, Urban and Natural Landscapes.” Science of the Total Environment 634: 1335–1345. 10.1016/j.scitotenv.2018.03.363. 29710633

[ece371986-bib-0012] Brühl, C. A. , S. Pieper , and B. Weber . 2011. “Amphibians at Risk? Susceptibility of Terrestrial Amphibian Life Stages to Pesticides.” Environmental Toxicology and Chemistry 30: 2465–2472. 10.1002/ETC.650. 21898550

[ece371986-bib-0013] Brühl, C. A. , T. Schmidt , S. Pieper , and A. Alscher . 2013. “Terrestrial Pesticide Exposure of Amphibians: An Underestimated Cause of Global Decline?” Scientific Reports 3: 1135. 10.1038/srep01135. 23350038 PMC3553602

[ece371986-bib-0014] Cai, M. , Y. Y. Li , M. Zhu , J. B. Li , and Z. F. Qin . 2020. “Evaluation of the Effects of Low Concentrations of Bisphenol AF on Gonadal Development Using the *Xenopus laevis* Model: A Finding of Testicular Differentiation Inhibition Coupled With Feminization.” Environmental Pollution 260: 113980. 10.1016/j.envpol.2020.113980. 31991354

[ece371986-bib-0015] Carr, J. A. , A. Gentles , E. E. Smith , et al. 2003. “Response of Larval *Xenopus laevis* to Atrazine: Assessment of Growth, Metamorphosis, and Gonadal and Laryngeal Morphology.” Environmental Toxicology and Chemistry 22: 396–405. 10.1002/etc.5620220222. 12558173

[ece371986-bib-0016] Chardard, D. , S. Kuntz , A. Chesnel , and S. Flament . 2003. “Effects of Androgens on Sex Differentiation of the Urodele *Pleurodeles waltl* .” Journal of Experimental Zoology Part A: Comparative Experimental Biology 296: 46–55. 10.1002/JEZ.A.10240. 12589690

[ece371986-bib-0017] Chen, C. Y. , K. M. Hathaway , and C. L. Folt . 2004. “Multiple Stress Effects of Vision Herbicide, pH, and Food on Zooplankton and Larval Amphibian Species From Forest Wetlands.” Environmental Toxicology and Chemistry 23: 823–831. 10.1897/03-108. 15095876

[ece371986-bib-0018] Cheong, S. , J.‐H. Yoo , S.‐R. Park , and H.‐C. Sung . 2013. “Age Estimation by Skeletochronology and Advertisement Call Variation in the Black‐Spotted Pond Frog ( *Rana nigromaculata* ).” Animal Cells and Systems 17: 141–146. 10.1080/19768354.2013.778215.

[ece371986-bib-0019] Chieffi, G. , G. Delrio , M. d'Istria , and M. A. Valentino . 1975. “Appearance of Sex Hormone Receptors in Frog ( *Rana esculenta* ) Tadpole Skin During Metamorphosis.” Experientia 31: 989–990. 10.1007/BF02358894. 169146

[ece371986-bib-0020] Chowdhury, S. , K. Gonzalez , M. Ç. K. Aytekin , et al. 2022. “Growth of Non‐English‐Language Literature on Biodiversity Conservation.” Conservation Biology 36: e13883. 10.1111/cobi.13883. 34981574 PMC9539909

[ece371986-bib-0021] Clotfelter, E. D. , A. M. Bell , and K. R. Levering . 2004. “The Role of Animal Behavior in the Study of Endocrine‐Disrupting Chemicals.” Animal Behaviour 68: 665–676. 10.1016/J.ANBEHAV.2004.05.004.

[ece371986-bib-0022] Coe, T. S. , P. B. Hamilton , D. Hodgson , et al. 2008. “An Environmental Estrogen Alters Reproductive Hierarchies, Disrupting Sexual Selection in Group‐Spawning Fish.” Environmental Science & Technology 42: 5020–5025. 10.1021/es800277q. 18678043

[ece371986-bib-0023] Colafrancesco, K. , and M. Gridi‐Papp . 2016. Vocal Sound Production and Acustic Communication in Amphibians and Reptiles, 51–82. Springer International Publishing. 10.1007/978-3-319-27721-9_3.

[ece371986-bib-0024] Coulter, D. P. , K. E. Huff Hartz , M. S. Sepúlveda , A. Godfrey , J. E. Garvey , and M. J. Lydy . 2019. “Lifelong Exposure to Dioxin‐Like PCBs Alters Paternal Offspring Care Behavior and Reduces Male Fish Reproductive Success.” Environmental Science & Technology 53: 11507–11514. 10.1021/acs.est.9b03460. 31369710

[ece371986-bib-0025] Daam, M. A. , P. Ilha , and L. Schiesari . 2020. “Acute Toxicity of Inorganic Nitrogen (Ammonium, Nitrate and Nitrite) to Tadpoles of Five Tropical Amphibian Species.” Ecotoxicology 29, no. 9: 1516–1521. 10.1007/s10646-020-02247-8. 32638180

[ece371986-bib-0026] de Cock, M. , Y. G. H. Maas , and M. van de Bor . 2012. “Does Perinatal Exposure to Endocrine Disruptors Induce Autism Spectrum and Attention Deficit Hyperactivity Disorders? Review.” Acta Paediatrica 101: 811–818. 10.1111/j.1651-2227.2012.02693.x. 22458970

[ece371986-bib-0027] Duan, R. Y. , and M. Y. Huang . 2016. “The Influence of Low‐Dose Cadmium on the Laryngeal Microstructure and Ultrastructure of *Pelophylax nigromaculata* .” Environmental Science and Pollution Research 23: 17322–17331. 10.1007/S11356-016-6942-4. 27225008

[ece371986-bib-0028] DuRant, S. E. , W. A. Hopkins , A. K. Davis , and L. M. Romero . 2015. “Evidence of Ectoparasite‐Induced Endocrine Disruption in an Imperiled Giant Salamander, the Eastern Hellbender (*Cryptobranchus alleganiensis*).” Journal of Experimental Biology 218: 2297–2304. 10.1242/JEB.118703. 26034123

[ece371986-bib-0029] Efosa, N. J. , W. Kleiner , W. Kloas , and F. Hoffmann . 2017. “Diclofenac Can Exhibit Estrogenic Modes of Action in Male *Xenopus laevis*, and Affects the Hypothalamus‐Pituitary‐Gonad Axis and Mating Vocalizations.” Chemosphere 173: 69–77. 10.1016/j.chemosphere.2017.01.030. 28107717

[ece371986-bib-0030] European Commission . 1996. European Workshop on the Impact of Endocrine Disrupters on Human Health and Wildlife, in: DG XII Weybridge Environment and Climate Research Programme Report Europe, 17549.

[ece371986-bib-0031] Feijó, M. , R. V. L. Martins , S. Socorro , L. Pereira , and S. Correia . 2021. “Effects of the Endocrine Disruptor Vinclozolin in Male Reproduction: A Systematic Review and Meta‐Analysis.” Biology of Reproduction 104, no. 5: 962–975. 10.1093/BIOLRE/IOAB018. 33524106

[ece371986-bib-0032] Filby, A. L. , G. C. Paull , F. Searle , M. Ortiz‐Zarragoitia , and C. R. Tyler . 2012. “Environmental Estrogen‐Induced Alterations of Male Aggression and Dominance Hierarchies in Fish: A Mechanistic Analysis.” Environmental Science & Technology 46: 3472–3479. 10.1021/es204023d. 22360147

[ece371986-bib-0033] Frątczak, M. , M. Kaczmarski , K. Szkudelska , and P. Tryjanowski . 2025. “Assessing Species Bias in Amphibian Research on Endocrine Disruptors: Beyond *Xenopus laevis* .” Frontiers in Environmental Science 13: 1–19. 10.3389/fenvs.2025.1556788.

[ece371986-bib-0034] Ghosh, A. , A. Tripathy , and D. Ghosh . 2022. “Impact of Endocrine Disrupting Chemicals (EDCs) on Reproductive Health of Human.” Proceedings of the Zoological Society 75: 16–30. 10.1007/S12595-021-00412-3.

[ece371986-bib-0035] González‐Alcaraz, M. N. , C. Malheiro , D. N. Cardoso , et al. 2020. “Bioaccumulation and Toxicity of Organic Chemicals in Terrestrial Invertebrates.” In Bioavailability of Organic Chemicals in Soil and Sediment, edited by J. J. Ortega‐Calvo and J. R. Parsons , 149–189. Springer International Publishing. 10.1007/698_2020_511.

[ece371986-bib-0036] Gore, A. C. , K. Krishnan , and M. P. Reilly . 2019. “Endocrine‐Disrupting Chemicals: Effects on Neuroendocrine Systems and the Neurobiology of Social Behavior.” Hormones and Behavior 111: 7–22. 10.1016/j.yhbeh.2018.11.006. 30476496 PMC6527472

[ece371986-bib-0037] Gosner, K. L. 1960. “A Simplified Table for Staging Anuran Embryos and Larvae With Notes on Identification.” Herpetologica 16: 183–190.

[ece371986-bib-0038] Goto, Y. , S. Kitamura , K. Kashiwagi , et al. 2006. “Suppression of Amphibian Metamorphosis by Bisphenol A and Related Chemical Substances.” Journal of Health Science 52: 160–168. 10.1248/jhs.52.160.

[ece371986-bib-0039] Greenberg, B. 1942. “Some Effects of Testosterone on the Sexual Pigmentation and Other Sex Characters of the Cricket Frog ( *Acris gryllus* ).” Journal of Experimental Zoology 91: 435–451. 10.1002/JEZ.1400910308.

[ece371986-bib-0040] Gyllenhammar, I. 2008. Amphibians Developmental Effects of Ethynylestradiol and Clotrimazole on the Reproductive System.

[ece371986-bib-0041] Hall, I. C. , and D. B. Kelley . 2021. “Endocrine Modulation of Acustic Communication: *Xenopus laevis* as a Model System.” In Neuroendocrine Regulation of Animal Vocalization: Mechanisms and Anthropogenic Factors in Animal Communication, 81–100. Elsevier. 10.1016/B978-0-12-815160-0.00009-8.

[ece371986-bib-0042] Halliday, T. R. 1990. “The Evoclution of Courtship Behavior in Newts and Salamanders.” In Advances in the Study of Behavior, edited by P. J. B. Slater , J. S. Rosenblatt , and C. Beer , 137–169. Academic Press. 10.1016/S0065-3454(08)60202-8.

[ece371986-bib-0043] Hayes, T. B. , A. Collins , M. Lee , et al. 2002. “Hermaphroditic, Demasculinized Frogs After Exposure to the Herbicide Atrazine at Low Ecologically Relevant Doses.” Proceedings of the National Academy of Sciences 99: 5476–5480. 10.1073/PNAS.082121499. PMC122794 11960004

[ece371986-bib-0139] Hayes, T. B. , V. Khoury , A. Narayan , et al. 2010. “Atrazine Induces Complete Feminization and Chemical Castration in Male African Clawed Frogs (*Xenopus laevis*).” Proceedings of the National Academy of Sciences of the United States of America 107, no. 10: 4612–4617. 10.1073/pnas.0909519107. 20194757 PMC2842049

[ece371986-bib-0044] Hayes, T. B. , and K. P. Menendez . 1999. “The Effect of Sex Steroids on Primary and Secondary Sex Differentiation in the Sexually Dichromatic Reedfrog ( *Hyperolius argus* : Hyperolidae) From the Arabuko Sokoke Forest of Kenya.” General and Comparative Endocrinology 115: 188–199. 10.1006/GCEN.1999.7321. 10417232

[ece371986-bib-0045] Hoffmann, F. , and W. Kloas . 2010. “An Environmentally Relevant Endocrine‐Disrupting Antiandrogen, Vinclozolin, Affects Calling Behavior of Male *Xenopus laevis* .” Hormones and Behavior 58: 653–659. 10.1016/j.yhbeh.2010.06.008. 20600051

[ece371986-bib-0046] Hoffmann, F. , and W. Kloas . 2012a. “Estrogens Can Disrupt Amphibian Mating Behavior.” PLoS One 7: e32097. 10.1371/JOURNAL.PONE.0032097. 22355410 PMC3280221

[ece371986-bib-0047] Hoffmann, F. , and W. Kloas . 2012b. “The Antiestrogens Tamoxifen and Fulvestrant Abolish Estrogenic Impacts of 17α‐Ethinylestradiol on Male Calling Behavior of *Xenopus laevis* .” PLoS One 7: e44715. 10.1371/JOURNAL.PONE.0044715. 23028589 PMC3445530

[ece371986-bib-0048] Hoffmann, F. , and W. Kloas . 2012c. “The Synthetic Progestogen, Levonorgestrel, but Not Natural Progesterone, Affects Male Mate Calling Behavior of *Xenopus laevis* .” General and Comparative Endocrinology 176, no. 3: 385–390. 10.1016/J.YGCEN.2012.02.009. 22391239

[ece371986-bib-0049] Hoffmann, F. , and W. Kloas . 2012d. “Effects of Environmentally Relevant Concentrations of the Xeno‐Androgen, Methyldihydrotestosterone, on Male and Female Mating Behavior in *Xenopus laevis* .” Chemosphere 87: 1246–1253. 10.1016/J.CHEMOSPHERE.2012.01.030. 22342339

[ece371986-bib-0050] Hoffmann, F. , and W. Kloas . 2016. “P,P′‐Dichlordiphenyldichloroethylene (p,P′‐DDE) Can Elicit Antiandrogenic and Estrogenic Modes of Action in the Amphibian *Xenopus laevis* .” Physiology and Behavior 167: 172–178. 10.1016/j.physbeh.2016.09.012. 27640133

[ece371986-bib-0051] Hoskins, T. D. , M. Dellapina , and M. D. Boone . 2017. “Short‐Term Atrazine Exposure at Breeding Has no Impact on Blanchard's Cricket Frog ( *Acris blanchardi* ) Reproductive Success.” Environmental Toxicology and Chemistry 36: 3284–3288. 10.1002/etc.3900. 28657116

[ece371986-bib-0052] Houck, L. D. , and N. L. Reagan . 1990. “Male Courtship Pheromones Increase Female Receptivity in a Plethodontid Salamander.” Animal Behaviour 39: 729–734. 10.1016/S0003-3472(05)80384-7.

[ece371986-bib-0053] Huang, M. Y. , R. Y. Duan , and X. Ji . 2015. “The Influence of Long‐Term Cadmium Exposure on Phonotaxis in Male *Pelophylax nigromaculata* .” Chemosphere 119: 763–768. 10.1016/J.CHEMOSPHERE.2014.08.014. 25192651

[ece371986-bib-0054] Hudecova, A. M. , K. E. A. Hansen , S. Mandal , et al. 2018. “A Human Exposure Based Mixture of Persistent Organic Pollutants Affects the Stress Response in Female Mice and Their Offspring.” Chemosphere 197: 585–593. 10.1016/j.chemosphere.2018.01.085. 29407821

[ece371986-bib-0055] Iglesias‐Carrasco, M. , M. L. Head , M. D. Jennions , J. Martín , and C. Cabido . 2017. “Leaf Extracts From an Exotic Tree Affect Responses to Chemical Cues in the Palmate Newt, *Lissotriton helveticus* .” Animal Behavior 127: 243–251. 10.1016/j.anbehav.2017.03.025.

[ece371986-bib-0056] Jagnytsch, O. , R. Opitz , I. Lutz , and W. Kloas . 2006. “Effects of Tetrabromobisphenol A on Larval Development and Thyroid Hormone‐Regulated Biomarkers of the Amphibian *Xenopus laevis* .” Environmental Research 101: 340–348. 10.1016/j.envres.2005.09.006. 16290818

[ece371986-bib-0057] Järup, L. , and A. Åkesson . 2009. “Current Status of Cadmium as an Environmental Health Problem.” Toxicology and Applied Pharmacology 238: 201–208. 10.1016/J.TAAP.2009.04.020. 19409405

[ece371986-bib-0058] Jürgens, M. D. , K. I. E. Holthaus , A. C. Johnson , J. L. Smith , M. Hetheridge , and R. J. Williams . 2002. “The Potential for Estradiol and Ethinylestradiol Degradation in English Rivers.” Environmental Toxicology and Chemistry 21: 480–488. 11883412

[ece371986-bib-0059] Kajta, M. , and A. K. Wójtowicz . 2013. “Impact of Endocrine‐Disrupting Chemicals on Neural Development and the Onset of Neurological Disorders.” Pharmacological Reports 65: 1632–1639. 10.1016/S1734-1140(13)71524-X. 24553011

[ece371986-bib-0060] Kikuyama, S. , K. Yamamoto , T. Iwata , and F. Toyoda . 2002. “Peptide and Protein Pheromones in Amphibians.” Comparative Biochemistry and Physiology, Part B: Biochemistry & Molecular Biology 132: 69–74. 10.1016/s1096-4959(01)00534-6. 11997210

[ece371986-bib-0062] Kloas, W. 2002. “Amphibians as a Model for the Study of Endocrine Disruptors.” International Review of Cytology 216: 1–57. 10.1016/s0074-7696(02)16002-5. 12049206

[ece371986-bib-0063] Kloas, W. , and I. Lutz . 2006. “Amphibians as Model to Study Endocrine Disrupters.” Journal of Chromatography A 1130: 16–27. 10.1016/j.chroma.2006.04.001. 16701677

[ece371986-bib-0064] Kloas, W. , R. Urbatzka , R. Opitz , et al. 2009. “Endocrine Disruption in Aquatic Vertebrates.” Annals of the New York Academy of Sciences 1163: 187–200. 10.1111/j.1749-6632.2009.04453.x. 19456339

[ece371986-bib-0065] Konno, K. , M. Akasaka , C. Koshida , et al. 2020. “Ignoring Non‐English‐Language Studies May Bias Ecological Meta‐Analyses.” Ecology and Evolution 10: 6373–6384. 10.1002/ece3.6368. 32724519 PMC7381574

[ece371986-bib-0066] La Merrill, M. A. , L. N. Vandenberg , M. T. Smith , et al. 2020. “Consensus on the Key Characteristics of Endocrine‐Disrupting Chemicals as a Basis for Hazard Identification.” Nature Reviews. Endocrinology 16: 45–57. 10.1038/s41574-019-0273-8. PMC6902641 31719706

[ece371986-bib-0067] LaFiandra, E. M. , K. J. Babbitt , and S. A. Sower . 2008. “Effects of Atrazine on Anuran Development Are Altered by the Presence of a Nonlethal Predator.” Journal of Toxicology and Environmental Health, Part A 71: 505–511. 10.1080/15287390801907442. 18338285

[ece371986-bib-0068] Lambert, M. R. , and D. K. Skelly . 2016. “Diverse Sources for Endocrine Disruption in the Wild.” Endocrine Disruptors 4: e1148803. 10.1080/23273747.2016.1148803.

[ece371986-bib-0069] Lange, I. G. , A. Daxenberger , B. Schiffer , H. Witters , D. Ibarreta , and H. H. D. Meyer . 2002. “Sex Hormones Originating From Different Livestock Production Systems: Fate and Potential Disrupting Activity in the Environment.” Analytica Chimica Acta 473, no. 1–2: 27–37. 10.1016/S0003-2670(02)00748-1.

[ece371986-bib-0070] Lefcort, H. , R. A. Meguire , L. H. Wilson , and W. F. Ettinger . 1998. “Heavy Metals Alter the Survival, Growth, Metamorphosis, and Antipredatory Behavior of Columbia Spotted Frog ( *Rana luteiventris* ) Tadpoles.” Archives of Environmental Contamination and Toxicology 35: 447–456. 10.1007/S002449900401. 9732476

[ece371986-bib-0071] Leininger, E. C. , and D. B. Kelley . 2015. “Evolution of Courtship Songs in Xenopus: Vocal Pattern Generation and Sound Production.” Cytogenetic and Genome Research 145: 302–314. 10.1159/000433483. 26138673

[ece371986-bib-0137] Li, X. , Y. Shen , B. Lang , J. Zhao , H. Wang , and Y. Zhang . 2021. “Influence of Octylphenol on Gene Expression of Gonadotropins and Their Receptors, Testicular Structure and Mating Behavior of Male *Rana chensinensis* .” Environmental Toxicology and Pharmacology 87: 103694. 10.1016/j.etap.2021.103694. 34153509

[ece371986-bib-0072] López‐Rodríguez, D. , C. F. Aylwin , V. Delli , et al. 2021. “Multi‐ and Transgenerational Outcomes of an Exposure to a Mixture of Endocrine‐Disrupting Chemicals (EDCs) on Puberty and Maternal Behavior in the Female Rat.” Environmental Health Perspectives 129: 087003. 10.1289/EHP8795. 34383603 PMC8360047

[ece371986-bib-0073] Luedtke, J. A. , J. Chanson , K. Neam , et al. 2023. “Ongoing Declines for the World's Amphibians in the Face of Emerging Threats.” Nature 622: 308–314. 10.1038/s41586-023-06578-4. 37794184 PMC10567568

[ece371986-bib-0074] Lutz, I. , W. Kloas , T. A. Springer , et al. 2008. “Development, Standardization and Refinement of Procedures for Evaluating Effects of Endocrine Active Compounds on Development and Sexual Differentiation of *Xenopus laevis* .” Analytical and Bioanalytical Chemistry 390: 2031–2048. 10.1007/S00216-008-1973-4. 18327572 PMC2287204

[ece371986-bib-0075] Markman, S. , I. A. Guschina , S. Barnsley , K. L. Buchanan , D. Pascoe , and C. T. Müller . 2007. “Endocrine Disrupting Chemicals Accumulate in Earthworms Exposed to Sewage Effluent.” Chemosphere 70: 119–125. 10.1016/j.chemosphere.2007.06.045. 17675209

[ece371986-bib-0077] Miodovnik, A. , S. M. Engel , C. Zhu , et al. 2011. “Endocrine Disruptors and Childhood Social Impairment.” Neurotoxicology 32: 261–267. 10.1016/j.neuro.2010.12.009. 21182865 PMC3057338

[ece371986-bib-0078] Miura, I. , H. Kitamoto , Y. Koizumi , M. Ogata , and K. Sasaki . 2011. “An X‐Linked Body Color Gene of the Frog Rana Rugosa and Its Application to the Molecular Analysis of Gonadal Sex Differentiation.” Sexual Development 5, no. 5: 250–258. 10.1159/000330365. 21832826

[ece371986-bib-0080] Mosconi, G. , O. Carnevali , M. F. Franzoni , et al. 2002. “Environmental Estrogens and Reproductive Biology in Amphibians.” General and Comparative Endocrinology 126, no. 2: 125–129. 10.1006/gcen.2002.7781. 12030767

[ece371986-bib-0081] Nieuwkoop, P. D. , and J. Faber . 2020. Normal Table of *Xenopus laevis* (Daudin): A Systematical and Chronological Survey of the Development From the Fertilized Egg Till the End of Metamorphosis. Garland Science. 10.1201/9781003064565.

[ece371986-bib-0082] Noriega, N. C. , and T. B. Hayes . 2000. “DDT Congener Effects on Secondary Sex Coloration in the Reed Frog *Hyperolius argus* : A Partial Evaluation of the *Hyperolius argus* Endocrine Screen.” Comparative Biochemistry and Physiology Part B: Biochemistry and Molecular Biology 126: 231–237. 10.1016/S0305-0491(00)00201-7. 10874170

[ece371986-bib-0083] Norris, D. O. , and J. A. Carr . 2013. Endocrine Disruption: Biological Bases for Health Effects in Wildlife and Humans. Oxford University Press.

[ece371986-bib-0084] Oka, T. , N. Mitsui , M. Hinago , et al. 2006. “All ZZ Male *Xenopus laevis* Provides a Clear Sex‐Reversal Test for Feminizing Endocrine Disruptors.” Ecotoxicology and Environmental Safety 63: 236–243. 10.1016/J.ECOENV.2005.07.018. 16139364

[ece371986-bib-0085] Orias, F. , S. Bony , A. Devaux , et al. 2015. “Tamoxifen Ecotoxicity and Resulting Risks for Aquatic Ecosystems.” Chemosphere 128: 79–84. 10.1016/J.CHEMOSPHERE.2015.01.002. 25666175

[ece371986-bib-0086] Oropesa, A. L. , and L. Guimarães . 2021. “Occurrence of Levonorgestrel in Water Systems and Its Effects on Aquatic Organisms: A Review.” Reviews of Environmental Contamination and Toxicology 254: 57–84. 10.1007/398_2020_44. 32494900

[ece371986-bib-0087] Ortiz‐Santaliestra, M. E. , A. Marco , M. Mari´ , M. J. Ferna'ndez , F. F.‐B. Itez , and M. Lizana . 2009. “Alteration of Courtship Behavior Because of Water Acidification and Minor Effect of Ammonium Nitrate in the Iberian Newt (*Lissotriton boscai*).” Environmental Toxicology and Chemistry 28: 1500–1505. 10.1897/08-580.1. 19220079

[ece371986-bib-0088] Orton, F. , B. Roberts‐Rhodes , C. Whatley , and C. R. Tyler . 2023. “A Review of Non‐Destructive Biomonitoring Techniques to Assess the Impacts of Pollution on Reproductive Health in Frogs and Toads.” Ecotoxicology and Environmental Safety 262: 115163. 10.1016/J.ECOENV.2023.115163. 37354567

[ece371986-bib-0089] Paetow, L. J. , R. I. Cue , B. D. Pauli , and D. J. Marcogliese . 2023. “Effects of Herbicides and the Chytrid Fungus Batrachochytrium Dendrobatidis on the Growth, Development and Survival of Larval American Toads ( *Anaxyrus americanus* ).” Ecotoxicology and Environmental Safety 259: 115021. 10.1016/J.ECOENV.2023.115021. 37216860

[ece371986-bib-0090] Papoulias, D. M. , M. S. Schwarz , and L. Mena . 2013. “Gonadal Abnormalities in Frogs (Lithobates spp.) Collected From Managed Wetlands in an Agricultural Region of Nebraska, USA.” Environmental Pollution 172: 1–8. 10.1016/J.ENVPOL.2012.07.042. 22982548

[ece371986-bib-0091] Park, D. , S. C. Hempleman , and C. R. Propper . 2001. “Endosulfan Exposure Disrupts Pheromonal Systems in the Red‐Spotted Newt: A Mechanism for Subtle Effects of Environmental Chemicals.” Environmental Health Perspectives 109: 669–673. 10.1289/EHP.01109669. 11485864 PMC1240369

[ece371986-bib-0092] Parrella, A. , M. Lavorgna , E. Criscuolo , C. Russo , and M. Isidori . 2014. “Estrogenic Activity and Cytotoxicity of Six Anticancer Drugs Detected in Water Systems.” Science of the Total Environment 485: 216–222. 10.1016/j.scitotenv.2014.03.050. 24727039

[ece371986-bib-0093] Patisaul, H. B. , and H. B. Adewale . 2009. “Long‐Term Effects of Environmental Endocrine Disruptors on Reproductive Physiology and Behavior.” Frontiers in Behavioral Neuroscience 3: 10. 10.3389/neuro.08.010.2009. 19587848 PMC2706654

[ece371986-bib-0094] Potter, K. A. , T. Bose , and A. Yamaguchi . 2005. “Androgen‐Induced Vocal Transformation in Adult Female African Clawed Frogs.” Journal of Neurophysiology 94, no. 1: 415–428. 10.1152/jn.01279.2004. 15758050

[ece371986-bib-0095] Poulsen, R. , N. Cedergreen , T. Hayes , and M. Hansen . 2018. “Nitrate: An Environmental Endocrine Disruptor? A Review of Evidence and Research Needs.” Environmental Science and Technology 52, no. 7: 3869–3887. 10.1021/acs.est.7b06419. 29494771

[ece371986-bib-0096] Quinnies, K. M. , T. J. Doyle , K. H. Kim , and E. F. Rissman . 2015. “Transgenerational Effects of Di‐(2‐Ethylhexyl) Phthalate (DEHP) on Stress Hormones and Behavior.” Endocrinology 156: 3077–3083. 10.1210/EN.2015-1326. 26168342 PMC4541619

[ece371986-bib-0097] Richards, C. M. 1982. “The Alteration of Chromatophore Expression by Sex Hormones in the Kenyan Reed Frog, *Hyperolius viridiflavus* .” General and Comparative Endocrinology 46: 59–67. 10.1016/0016-6480(82)90163-0. 7060936

[ece371986-bib-0098] Robinson, C. E. , C. K. Elvidge , R. A. Frank , et al. 2023. “Naphthenic Acid Fraction Compounds Reduce the Reproductive Success of Wood Frogs (*Rana sylvatica*) by Affecting Offspring Viability.” Environmental Pollution 316: 120455. 10.1016/j.envpol.2022.120455. 36270565

[ece371986-bib-0099] Rohr, J. R. , and K. A. McCoy . 2010. “A Qualitative Meta‐Analysis Reveals Consistent Effects of Atrazine on Freshwater Fish and Amphibians.” Environmental Health Perspectives 118: 20–32. 10.1289/EHP.0901164. 20056568 PMC2831963

[ece371986-bib-0100] Rojas, B. , J. P. Lawrence , and R. Márquez . 2023. “Amcphibian Coloration: Proximate Mechanisms, Function, and Evolution.” In Evolutionary Ecology of Amphibians. CRC Press.

[ece371986-bib-0138] Rollmann, S. M. , L. D. Houck , and R. C. Feldhoff . 1999. “Proteinaceous Pheromone Affecting Female Receptivity in a Terrestrial Salamander.” Science 285, no. 5435: 1907–1909. 10.1126/science.285.5435.1907. 10489368

[ece371986-bib-0101] Rosenfeld, C. S. 2015. “Bisphenol A and Phthalate Endocrine Disruption of Parental and Social Behaviors.” Frontiers in Neuroscience 9: 9. 10.3389/fnins.2015.00057. 25784850 PMC4347611

[ece371986-bib-0102] Rozenblut‐Kościsty, B. , M. Ogielska , J. Hahn , et al. 2019. “Impacts of the Synthetic Androgen Trenbolone on Gonad Differentiation and Development—Comparisons Between Three Deeply Diverged Anuran Families.” Scientific Reports 9: 1–15. 10.1038/s41598-019-45985-4. 31270347 PMC6610071

[ece371986-bib-0103] Saaristo, M. , J. A. Craft , K. K. Lehtonen , and K. Lindström . 2010. “An Endocrine Disrupting Chemical Changes Courtship and Parental Care in the Sand Goby.” Aquatic Toxicology 97: 285–292. 10.1016/j.aquatox.2009.12.015. 20060601

[ece371986-bib-0104] Saaristo, M. , C. P. Johnstone , K. Xu , M. Allinson , and B. B. M. Wong . 2019. “The Endocrine Disruptor, 17α‐Ethinyl Estradiol, Alters Male Mate Choice in a Freshwater Fish.” Aquatic Toxicology 208: 118–125. 10.1016/j.aquatox.2019.01.006. 30658282

[ece371986-bib-0105] Säfholm, M. , A. Ribbenstedt , J. Fick , and C. Berg . 2014. “Risks of Hormonally Active Pharmaceuticals to Amphibians: A Growing Concern Regarding Progestagens.” Philosophical Transactions of the Royal Society, B: Biological Sciences 369: 20130577. 10.1098/rstb.2013.0577. PMC4213589 25405966

[ece371986-bib-0106] Scaia, M. F. , L. S. de Gregorio , L. Franco‐Belussi , M. Succi‐Domingues , and C. de Oliveira . 2019. “Gonadal, Body Color, and Genotoxic Alterations in *Lithobates catesbeianus* Tadpoles Exposed to Nonylphenol.” Environmental Science and Pollution Research 26: 22209–22219. 10.1007/S11356-019-05403-8. 31152429

[ece371986-bib-0107] Schiesari, L. , B. Grillitsch , and H. Grillitsch . 2007. “Biogeographic Biases in Research and Their Consequences for Linking Amphibian Declines to Pollution.” Conservation Biology 21, no. 2: 465–471. 10.1111/j.1523-1739.2006.00616.x. 17391196

[ece371986-bib-0108] Schmidt, R. S. 1966. “Hormonal Mechanisms of Frog Mating Calling.” Copeia 1966: 637–644. 10.2307/1441395.

[ece371986-bib-0109] Schwendiman, A. L. , and C. R. Propper . 2012. “A Common Environmental Contaminant Affects Sexual Behavior in the Clawed Frog, *Xenopus tropicalis* .” Physiology & Behavior 106: 520–526. 10.1016/j.physbeh.2012.03.035. 22504493

[ece371986-bib-0110] Secondi, J. , E. Hinot , Z. Djalout , S. Sourice , and A. Jadas‐Hécart . 2009. “Realistic Nitrate Concentration Alters the Expression of Sexual Traits and Olfactory Male Attractiveness in Newts.” Functional Ecology 23: 800–808. 10.1111/J.1365-2435.2009.01558.X.

[ece371986-bib-0111] Secondi, J. , V. Lepetz , G. Cossard , and S. Sourice . 2013. “Nitrate Affects Courting and Breathing but Not Escape Performance in Adult Newts.” Behavioral Ecology and Sociobiology 67: 1757–1765. 10.1007/S00265-013-1583-9.

[ece371986-bib-0112] Sever, D. M. , and N. L. Staub . 2011. “Hormones, Sex Accessory Structures, and Secondary Sexual Characteristics in Amphibians.” In Hormones and Reproduction of Vertebrates, 83–98. Academic Press. 10.1016/B978-0-12-374931-4.10005-7.

[ece371986-bib-0113] Sievers, M. , R. Hale , K. M. Parris , S. D. Melvin , C. M. Lanctôt , and S. E. Swearer . 2019. “Contaminant‐Induced Behavior al Changes in Amphibians: A Meta‐Analysis.” Science of the Total Environment 693: 133570. 10.1016/J.SCITOTENV.2019.07.376. 31369889

[ece371986-bib-0115] Silva, M. H. , and D. Gammon . 2009. “An Assessment of the Developmental, Reproductive, and Neurotoxicity of Endosulfan.” Birth Defects Research Part B: Developmental and Reproductive Toxicology 86, no. 1: 1–28. 10.1002/BDRB.20183. 19243027

[ece371986-bib-0116] Söffker, M. , and C. R. Tyler . 2012. “Endocrine Disrupting Chemicals and Sexual Behaviors in Fish—A Critical Review on Effects and Possible Consequences.” Critical Reviews in Toxicology 42: 653–668. 10.3109/10408444.2012.692114. 22697575

[ece371986-bib-0117] Swanson, J. E. , E. Muths , C. L. Pierce , et al. 2018. “Exploring the Amphibian Exposome in an Agricultural Landscape Using Telemetry and Passive Sampling.” Scientific Reports 8: 1–10. 10.1038/s41598-018-28132-3. 29968741 PMC6030078

[ece371986-bib-0118] Tamschick, S. , B. Rozenblut‐Kościsty , M. Ogielska , et al. 2016. “The Plasticizer Bisphenol A Affects Somatic and Sexual Development, but Differently in Pipid, Hylid and Bufonid Anurans.” Eng145vironmental Pollution 216: 282–291. 10.1016/j.envpol.2016.05.091. 27285164

[ece371986-bib-0119] Tamschick, S. , B. Rozenblut‐Kościsty , M. Ogielska , et al. 2016. “Impaired Gonadal and Somatic Development Corroborate Vulnerability Differences to the Synthetic Estrogen Ethinylestradiol Among Deeply Diverged Anuran Lineages.” Aquatic Toxicology 177: 503–514. 10.1016/J.AQUATOX.2016.07.001. 27434076

[ece371986-bib-0120] Thomas, K. V. , M. R. Hurst , P. Matthiessen , M. McHugh , A. Smith , and M. J. Waldock . 2002. “An Assessment of In Vitro Androgenic Activity and the Identification of Environmental Androgens in United Kingdom Estuaries.” Environmental Toxicology and Chemistry 21, no. 7: 1456–1461. 10.1002/ETC.5620210717. 12109746

[ece371986-bib-0122] Tomkins, P. , M. Saaristo , M. G. Bertram , M. Michelangeli , R. B. Tomkins , and B. B. M. Wong . 2018. “An Endocrine‐Disrupting Agricultural Contaminant Impacts Sequential Female Mate Choice in Fish.” Environmental Pollution 237: 103–110. 10.1016/j.envpol.2018.02.046. 29477864

[ece371986-bib-0124] Ujhegyi, N. , and V. Bókony . 2020. “Skin Coloration as a Possible Non‐Invasive Marker for Skewed Sex Ratios and Gonadal Abnormalities in Immature Common Toads (*Bufo bufo*).” Ecological Indicators 113: 106175. 10.1016/J.ECOLIND.2020.106175.

[ece371986-bib-0125] Uzendoski, K. , E. Maksymovitch , and P. Verrell . 1993. “Do the Risks of Predation and Intermale Competition Affect Courtship Behavior in the Salamander *Desmognathus ochrophaeus*?” Behavioral Ecology and Sociobiology 32: 421–427. 10.1007/BF00168826.

[ece371986-bib-0126] Wake, D. B. , and M. S. Koo . 2018. “Amphibians.” Current Biology 28: R1237–R1241. 10.1016/j.cub.2018.09.028. 30399342

[ece371986-bib-0127] Walkowiak, W. 2006. “Call Production and Neural Basis of Vocalization.” In Hearing and Sound Communication in Amphibians, Springer Handbook of Auditory Research, edited by P. M. Narins , A. S. Feng , R. R. Fay , and A. N. Popper , 87–112. Springer. 10.1007/978-0-387-47796-1_4.

[ece371986-bib-0128] Wells, K. D. 2008. The Ecology and Behavior of Amphibians. University of Chicago Press.

[ece371986-bib-0129] Wells, K. D. , and J. J. Schwartz . 2006. “The Behavioral Ecology of Anuran Communication.” In Hearing and Sound Communication in Amphibians, Springer Handbook of Auditory Research, edited by P. M. Narins , A. S. Feng , R. R. Fay , and A. N. Popper , 44–86. Springer. 10.1007/978-0-387-47796-1_3.

[ece371986-bib-0130] Wise, A. , K. O'Brien , and T. Woodruff . 2010. “Are Oral Contraceptives a Significant Contributor to the Estrogenicity of Drinking Water?” Environmental Science & Technology 45, no. 1: 51–60. 10.1021/ES1014482. 20977246

[ece371986-bib-0131] Wojtaszek, B. F. , B. Staznik , D. T. Chartrand , G. R. Stephenson , and D. G. Thompson . 2004. “Effects of Vision Herbicide on Mortality, Avoidance Response, and Growth of Amphibian Larvae in Two Forest Wetlands.” Environmental Toxicology and Chemistry 23: 832–842. 10.1897/02-281. 15095877

[ece371986-bib-0133] Woodley, S. 2015. “Chemosignals, Hormones, and Amphibian Reproduction. Hormones and Behavior, Chemosignals and Reproduction.” 68: 3–13. 10.1016/j.yhbeh.2014.06.008. 24945995

[ece371986-bib-0134] Woodley, S. K. 2011. “Hormones and Reproductive Behavior in Amphibians.” In Hormones and Reproduction of Vertebrates, 143–169. Academic Press. 10.1016/B978-0-12-374931-4.10008-2.

[ece371986-bib-0135] Zhang, W. S. , E. J. Farmer , D. Muhanzi , and V. L. Trudeau . 2022. “Petroleum‐Derived Naphthenic Acids Disrupt Hormone‐Dependent Sexual Behavior s in Male Western Clawed Frogs.” Conservation Physiology 10: coac030. 10.1093/CONPHYS/COAC030. 35602560 PMC9115893

[ece371986-bib-0136] Zornik, E. , and A. Yamaguchi . 2008. “Sexually Differentiated Central Pattern Generators in *Xenopus laevis* .” Trends in Neurosciences 31, no. 6: 296–302. 10.1016/j.tins.2008.03.001. 18471902 PMC2575109

